# Supplementary material for: Normative evidence weighing and accumulation in correlated environments
Source: eLife. 2025 Jul 14;13:RP100258. doi: 10.7554/eLife.100258 (PMC12259025; doi:10.7554/eLife.100258)
Supplement: Supplementary file 1. [file elife-100258-supp1.docx]

**Supplementary file 1 for:**

Tardiff, N., Kang, J., & Gold, J. I. (2024). Normative evidence weighing and accumulation in correlated environments. *eLife,* ***13***:RP100258.

| \|ρ\| | parameter | mean | SD | SEM |
| --- | --- | --- | --- | --- |
| 0.2 | *B_0_* | 1.775 | 0.513 | 0.103 |
|  | *t_B_* | 0.128 | 0.123 | 0.025 |
|  | *k_0_* | 17.458 | 3.405 | 0.681 |
|  | *ndt* | 0.328 | 0.162 | 0.032 |
|  | *𝜆* | 0.011 | 0.008 | 0.002 |
| 0.4 | *B_0_* | 1.911 | 0.644 | 0.129 |
|  | *t_B_* | 0.131 | 0.109 | 0.022 |
|  | *k_0_* | 19.543 | 4.723 | 0.945 |
|  | *ndt* | 0.399 | 0.284 | 0.057 |
|  | *𝜆* | 0.013 | 0.018 | 0.004 |
| 0.6 | *B_0_* | 1.792 | 0.556 | 0.111 |
|  | *t_B_* | 0.129 | 0.108 | 0.022 |
|  | *k_0_* | 18.021 | 3.302 | 0.66 |
|  | *ndt* | 0.264 | 0.11 | 0.022 |
|  | *𝜆* | 0.049 | 0.044 | 0.009 |
| 0.8 | *B_0_* | 1.779 | 0.49 | 0.098 |
|  | *t_B_* | 0.214 | 0.13 | 0.026 |
|  | *k_0_* | 20.304 | 3.089 | 0.618 |
|  | *ndt* | 0.359 | 0.171 | 0.034 |
|  | *𝜆* | 0.119 | 0.049 | 0.01 |

**Supplementary file 1a.** *Base* model average best-fitting parameter values.

| \|ρ\| | parameter | mean | SD | SEM |
| --- | --- | --- | --- | --- |
| 0.2 | *B_0_* | 1.772 | 0.504 | 0.101 |
|  | *t_B_* | 0.128 | 0.121 | 0.024 |
|  | *k_0_* | 17.382 | 3.73 | 0.746 |
|  | *k_−_* | 18.122 | 3.551 | 0.71 |
|  | *k_+_* | 17.169 | 3.529 | 0.706 |
|  | *ndt* | 0.332 | 0.17 | 0.034 |
|  | *𝜆* | 0.011 | 0.009 | 0.002 |
| 0.4 | *B_0_* | 1.942 | 0.669 | 0.134 |
|  | *t_B_* | 0.135 | 0.116 | 0.023 |
|  | *k_0_* | 19.081 | 4.693 | 0.939 |
|  | *k_−_* | 22.008 | 5.515 | 1.103 |
|  | *k_+_* | 17.75 | 4.109 | 0.822 |
|  | *ndt* | 0.392 | 0.273 | 0.055 |
|  | *𝜆* | 0.013 | 0.017 | 0.003 |
| 0.6 | *B_0_* | 1.818 | 0.566 | 0.113 |
|  | *t_B_* | 0.127 | 0.108 | 0.022 |
|  | *k_0_* | 17.122 | 3.123 | 0.625 |
|  | *k_−_* | 21.592 | 4.125 | 0.825 |
|  | *k_+_* | 15.932 | 2.926 | 0.585 |
|  | *ndt* | 0.248 | 0.115 | 0.023 |
|  | *𝜆* | 0.05 | 0.045 | 0.009 |
| 0.8 | *B_0_* | 1.843 | 0.477 | 0.095 |
|  | *t_B_* | 0.19 | 0.127 | 0.025 |
|  | *k_0_* | 18.43 | 2.662 | 0.532 |
|  | *k_−_* | 29.082 | 8.611 | 1.722 |
|  | *k_+_* | 17.301 | 2.703 | 0.541 |
|  | *ndt* | 0.316 | 0.19 | 0.038 |
|  | *𝜆* | 0.115 | 0.047 | 0.009 |

**Supplementary file 1b.** *Drift* model average best-fitting parameter values.

| \|ρ\| | parameter | mean | SD | SEM |
| --- | --- | --- | --- | --- |
| 0.2 | *B_0_* | 1.766 | 0.528 | 0.106 |
|  | $\hat{\rho}_{B-}$ | −0.131 | 0.085 | 0.017 |
|  | $\hat{\rho}_{B+}$ | 0.108 | 0.111 | 0.022 |
|  | *t_B_* | 0.127 | 0.131 | 0.026 |
|  | *k_0_* | 17.66 | 3.69 | 0.738 |
|  | *ndt* | 0.336 | 0.164 | 0.033 |
|  | *𝜆* | 0.012 | 0.009 | 0.002 |
| 0.4 | *B_0_* | 1.948 | 0.702 | 0.14 |
|  | $\hat{\rho}_{B-}$ | −0.285 | 0.088 | 0.018 |
|  | $\hat{\rho}_{B+}$ | 0.245 | 0.149 | 0.03 |
|  | *t_B_* | 0.124 | 0.114 | 0.023 |
|  | *k_0_* | 19.244 | 4.762 | 0.952 |
|  | *ndt* | 0.402 | 0.282 | 0.056 |
|  | *𝜆* | 0.01 | 0.011 | 0.002 |
| 0.6 | *B_0_* | 1.796 | 0.51 | 0.102 |
|  | $\hat{\rho}_{B-}$ | −0.484 | 0.07 | 0.014 |
|  | $\hat{\rho}_{B+}$ | 0.443 | 0.158 | 0.032 |
|  | *t_B_* | 0.087 | 0.093 | 0.019 |
|  | *k_0_* | 16.972 | 3.263 | 0.653 |
|  | *ndt* | 0.267 | 0.126 | 0.025 |
|  | *𝜆* | 0.009 | 0.007 | 0.001 |
| 0.8 | *B_0_* | 1.856 | 0.48 | 0.096 |
|  | $\hat{\rho}_{B-}$ | −0.685 | 0.065 | 0.013 |
|  | $\hat{\rho}_{B+}$ | 0.493 | 0.137 | 0.027 |
|  | *t_B_* | 0.106 | 0.099 | 0.02 |
|  | *k_0_* | 17.1 | 2.77 | 0.554 |
|  | *ndt* | 0.285 | 0.179 | 0.036 |
|  | *𝜆* | 0.013 | 0.014 | 0.003 |

**Supplementary file 1c.** *Bound-*$\hat{\rho}$ model average best-fitting parameter values.

| \|ρ\| | parameter | mean | SD | SEM |
| --- | --- | --- | --- | --- |
| 0.2 | *B_0_* | 1.777 | 0.53 | 0.106 |
|  | $\hat{\rho}_{-}$ | −0.15 | 0.055 | 0.011 |
|  | $\hat{\rho}_{+}$ | 0.132 | 0.08 | 0.016 |
|  | *t_B_* | 0.126 | 0.124 | 0.025 |
|  | *k_0_* | 17.488 | 3.522 | 0.704 |
|  | *ndt* | 0.326 | 0.164 | 0.033 |
|  | *𝜆* | 0.011 | 0.01 | 0.002 |
| 0.4 | *B_0_* | 1.967 | 0.692 | 0.138 |
|  | $\hat{\rho}_{-}$ | −0.317 | 0.066 | 0.013 |
|  | $\hat{\rho}_{+}$ | 0.268 | 0.08 | 0.016 |
|  | *t_B_* | 0.128 | 0.112 | 0.022 |
|  | *k_0_* | 19.401 | 4.981 | 0.996 |
|  | *ndt* | 0.39 | 0.29 | 0.058 |
|  | *𝜆* | 0.008 | 0.007 | 0.001 |
| 0.6 | *B_0_* | 1.803 | 0.505 | 0.101 |
|  | $\hat{\rho}_{-}$ | −0.487 | 0.05 | 0.01 |
|  | $\hat{\rho}_{+}$ | 0.445 | 0.123 | 0.025 |
|  | *t_B_* | 0.089 | 0.098 | 0.02 |
|  | *k_0_* | 17.62 | 3.639 | 0.728 |
|  | *ndt* | 0.272 | 0.111 | 0.022 |
|  | *𝜆* | 0.011 | 0.016 | 0.003 |
| 0.8 | *B_0_* | 1.822 | 0.429 | 0.086 |
|  | $\hat{\rho}_{-}$ | −0.697 | 0.052 | 0.01 |
|  | $\hat{\rho}_{+}$ | 0.536 | 0.139 | 0.028 |
|  | *t_B_* | 0.097 | 0.095 | 0.019 |
|  | *k_0_* | 18.182 | 3.094 | 0.619 |
|  | *ndt* | 0.305 | 0.175 | 0.035 |
|  | *𝜆* | 0.011 | 0.014 | 0.003 |

**Supplementary file 1d.** *Full-*$\hat{\rho}$ model average best-fitting parameter values.

| \|ρ\| | parameter | mean | SD | SEM |
| --- | --- | --- | --- | --- |
| 0.2 | *B_0_* | 1.795 | 0.529 | 0.106 |
|  | $\hat{\rho}_{B-}$ | −0.098 | 0.088 | 0.018 |
|  | $\hat{\rho}_{B+}$ | 0.161 | 0.14 | 0.028 |
|  | *t_B_* | 0.13 | 0.126 | 0.025 |
|  | *k_0_* | 17.898 | 3.808 | 0.762 |
|  | $\hat{\rho}_{SD-}$ | −0.013 | 0.256 | 0.051 |
|  | $\hat{\rho}_{SD+}$ | 0.222 | 0.284 | 0.057 |
|  | *ndt* | 0.324 | 0.162 | 0.032 |
|  | *𝜆* | 0.013 | 0.011 | 0.002 |
| 0.4 | *B_0_* | 1.959 | 0.701 | 0.14 |
|  | $\hat{\rho}_{B-}$ | −0.298 | 0.083 | 0.017 |
|  | $\hat{\rho}_{B+}$ | 0.351 | 0.121 | 0.024 |
|  | *t_B_* | 0.13 | 0.118 | 0.024 |
|  | *k_0_* | 19.717 | 4.745 | 0.949 |
|  | $\hat{\rho}_{SD-}$ | −0.276 | 0.155 | 0.031 |
|  | $\hat{\rho}_{SD+}$ | 0.443 | 0.277 | 0.055 |
|  | *ndt* | 0.4 | 0.279 | 0.056 |
|  | *𝜆* | 0.011 | 0.012 | 0.002 |
| 0.6 | *B_0_* | 1.82 | 0.535 | 0.107 |
|  | $\hat{\rho}_{B-}$ | −0.431 | 0.078 | 0.016 |
|  | $\hat{\rho}_{B+}$ | 0.452 | 0.101 | 0.02 |
|  | *t_B_* | 0.091 | 0.092 | 0.018 |
|  | *k_0_* | 17.918 | 3.651 | 0.73 |
|  | $\hat{\rho}_{SD-}$ | −0.328 | 0.161 | 0.032 |
|  | $\hat{\rho}_{SD+}$ | 0.464 | 0.24 | 0.048 |
|  | *ndt* | 0.26 | 0.109 | 0.022 |
|  | *𝜆* | 0.012 | 0.014 | 0.003 |
| 0.8 | *B_0_* | 1.87 | 0.471 | 0.094 |
|  | $\hat{\rho}_{B-}$ | −0.677 | 0.07 | 0.014 |
|  | $\hat{\rho}_{B+}$ | 0.483 | 0.16 | 0.032 |
|  | *t_B_* | 0.109 | 0.1 | 0.02 |
|  | *k_0_* | 18.066 | 3.262 | 0.652 |
|  | $\hat{\rho}_{SD-}$ | −0.623 | 0.162 | 0.032 |
|  | $\hat{\rho}_{SD+}$ | 0.41 | 0.262 | 0.052 |
|  | *ndt* | 0.286 | 0.177 | 0.035 |
|  | *𝜆* | 0.013 | 0.015 | 0.003 |

**Supplementary file 1e.** *Scaled-*$\hat{\rho}$ model average best-fitting parameter values.

| \|ρ\| | parameter | mean | SD | SEM |
| --- | --- | --- | --- | --- |
| 0.2 | *B_0_* | 1.766 | 0.525 | 0.105 |
|  | $\hat{\rho}_{B-}$ | −0.126 | 0.112 | 0.022 |
|  | $\hat{\rho}_{B+}$ | 0.145 | 0.127 | 0.025 |
|  | *t_B_* | 0.127 | 0.126 | 0.025 |
|  | *k_0_* | 17.149 | 3.28 | 0.656 |
|  | *k_−_* | 17.36 | 3.666 | 0.733 |
|  | *k_+_* | 18.186 | 3.631 | 0.726 |
|  | *ndt* | 0.341 | 0.167 | 0.033 |
|  | *𝜆* | 0.012 | 0.009 | 0.002 |
| 0.4 | *B_0_* | 1.931 | 0.705 | 0.141 |
|  | $\hat{\rho}_{B-}$ | −0.315 | 0.095 | 0.019 |
|  | $\hat{\rho}_{B+}$ | 0.275 | 0.146 | 0.029 |
|  | *t_B_* | 0.123 | 0.116 | 0.023 |
|  | *k_0_* | 19.551 | 4.923 | 0.985 |
|  | *k_−_* | 18.644 | 4.833 | 0.967 |
|  | *k_+_* | 19.676 | 4.531 | 0.906 |
|  | *ndt* | 0.419 | 0.268 | 0.054 |
|  | *𝜆* | 0.01 | 0.008 | 0.002 |
| 0.6 | *B_0_* | 1.829 | 0.547 | 0.109 |
|  | $\hat{\rho}_{B-}$ | −0.518 | 0.072 | 0.014 |
|  | $\hat{\rho}_{B+}$ | 0.419 | 0.193 | 0.039 |
|  | *t_B_* | 0.092 | 0.098 | 0.02 |
|  | *k_0_* | 17.547 | 3.283 | 0.657 |
|  | *k_−_* | 14.911 | 3.372 | 0.674 |
|  | *k_+_* | 18.446 | 4.074 | 0.815 |
|  | *ndt* | 0.27 | 0.104 | 0.021 |
|  | *𝜆* | 0.011 | 0.01 | 0.002 |
| 0.8 | *B_0_* | 1.856 | 0.484 | 0.097 |
|  | $\hat{\rho}_{B-}$ | −0.703 | 0.05 | 0.01 |
|  | $\hat{\rho}_{B+}$ | 0.554 | 0.154 | 0.031 |
|  | *t_B_* | 0.106 | 0.098 | 0.02 |
|  | *k_0_* | 18.572 | 3.459 | 0.692 |
|  | *k_−_* | 14.138 | 2.881 | 0.576 |
|  | *k_+_* | 20.279 | 3.632 | 0.726 |
|  | *ndt* | 0.298 | 0.166 | 0.033 |
|  | *𝜆* | 0.013 | 0.015 | 0.003 |

**Supplementary file 1f.** *Bound-*$\hat{\rho}$ *+ drift* model average best-fitting parameter values.
